# A computational biomarker of juvenile myoclonic epilepsy from resting-state MEG

**DOI:** 10.1016/j.clinph.2020.12.021

**Published:** 2021-04

**Authors:** Marinho A. Lopes, Dominik Krzemiński, Khalid Hamandi, Krish D. Singh, Naoki Masuda, John R. Terry, Jiaxiang Zhang

**Affiliations:** a Cardiff University Brain Research Imaging Centre, School of Psychology, Cardiff University, Cardiff CF24 4HQ, United Kingdom; b The Welsh Epilepsy Unit, Department of Neurology, University Hospital of Wales, Cardiff CF14 4XW, United Kingdom; c Department of Mathematics, University at Buffalo, State University of New York, USA; d Computational and Data-Enabled Science and Engineering Program, University at Buffalo, State University of New York, USA; e EPSRC Centre for Predictive Modelling in Healthcare, University of Birmingham, Birmingham, United Kingdom; f Centre for Systems Modelling and Quantitative Biomedicine, University of Birmingham, Edgbaston, United Kingdom; g Institute for Metabolism and Systems Research, University of Birmingham, Edgbaston, United Kingdom

**Keywords:** Epilepsy diagnosis, Juvenile myoclonic epilepsy, Biomarker, MEG, Functional connectivity, Phenomenological model

## Abstract

**Objective:**

For people with idiopathic generalized epilepsy, functional networks derived from their resting-state scalp electrophysiological recordings have shown an inherent higher propensity to generate seizures than those from healthy controls when assessed using the concept of brain network ictogenicity (BNI). Herein we tested whether the BNI framework is applicable to resting-state magnetoencephalography (MEG) from people with juvenile myoclonic epilepsy (JME).

**Methods:**

The BNI framework consists in deriving a functional network from apparently normal brain activity, placing a mathematical model of ictogenicity into the network and then computing how often such network generates seizures in silico. We considered data from 26 people with JME and 26 healthy controls.

**Results:**

We found that resting-state MEG functional networks from people with JME are characterized by a higher propensity to generate seizures (i.e., higher BNI) than those from healthy controls. We found a classification accuracy of 73%.

**Conclusions:**

The BNI framework is applicable to MEG and was capable of differentiating people with epilepsy from healthy controls.

**Significance:**

The BNI framework may be applied to resting-state MEG to aid in epilepsy diagnosis.

## Highlights

• Computational modelling is combined with MEG to differentiate people with juvenile myoclonic epilepsy from healthy controls.
• Brain network ictogenicity (BNI) was found higher in people with juvenile myoclonic epilepsy relative to healthy controls.
• BNI’s classification accuracy in our cohort was 73%.

## 1 Introduction

Epilepsy is one of the most common neurological disorders with an estimated 5 million new diagnosis each year (WHO, 2019). The diagnosis of epilepsy is based on clinical history and supported by clinical electroencephalography (EEG). The presence of interictal spikes in the routine scalp EEG recordings is one of the most valuable biomarkers of epilepsy (Pillai and Sperling, 2006). However, the presence of interictal epileptiform discharges (IED) in a routine EEG is low, ranging between 25 and 56% (Smith, 2005, Benbadis et al., 2020). Furthermore, about 10% of people with epilepsy do not show IEDs even after repeated or prolonged EEG (Smith, 2005, Benbadis et al., 2020). On the other hand, specificity is also suboptimal, ranging between 78 and 98% (Smith, 2005), which, for example, may delay the diagnosis of psychogenic nonepileptic attacks by 7 to 10 years (Benbadis, 2009).

The low sensitivity of IEDs results from IEDs being typically rare events. This may be a consequence of their sources being deep in the brain and/or the extent of cortex involved in epileptic activity being undetectable at the scalp surface (Pillai and Sperling, 2006). Consequently, much of the routine clinical EEG recording consists of brain activity that appears normal to visual inspection, which without other visible disturbances in background rhythms is considered non-informative. However, growing evidence suggests that such sections of interictal EEG without IEDs may be used to inform epilepsy diagnosis (e.g. Larsson and Kostov, 2005, Schmidt et al., 2016, Verhoeven et al., 2018). Larsson and Kostov (2005) showed that there is a shift in the peak of the alpha power towards lower frequencies in interictal EEG from people with both focal and generalized epilepsy. More recently, Abela et al. (2019) found that a slower alpha rhythm may be an indicator of seizure liability. Other studies have used graph theory to test whether functional networks derived from interictal EEG differ from EEG obtained from healthy controls. Functional networks are graphs, where nodes correspond to brain regions and connections are inferred from statistical dependencies (e.g. correlations) between brain signals from the regions. Such networks may represent the brain’s functional coordination between regions (Park and Friston, 2013, Bassett and Sporns, 2017). It has been found that functional networks from people with epilepsy are more “regular” (i.e. higher path lengths between nodes) and deviate more from small-world structures than those found in healthy controls (Horstmann et al., 2010, Quraan et al., 2013). Functional network alterations inferred from resting-state EEG have also been used to differentiate children with focal epilepsy from healthy children (van Diessen et al., 2013, van Diessen et al., 2016). Furthermore, resting-state EEG functional networks from people with idiopathic generalized epilepsy (IGE) (also known as genetic generalised epilepsy) were shown to have more functional connections than healthy controls (Chowdhury et al., 2014). Functional networks inferred from interictal EEG from people with temporal lobe epilepsy have also been shown to differ from those from healthy controls (Coito et al., 2016).

All these studies show that functional networks based on apparently normal EEG may aid in the diagnosis of epilepsy. However, these studies lack mechanistic insights as to why such differences may be related to epilepsy. To build such understanding, we and others have proposed to use mathematical models of epilepsy to assess the functional networks and elucidate as to why a brain may be prone to generate seizures (Schmidt et al., 2014, Schmidt et al., 2016, Petkov et al., 2014, Lopes et al., 2019). In particular, resting-state EEG functional networks from people with IGE were shown to be more prone to support synchronization phenomena and the emergence of seizure-like activity than those from controls (Schmidt et al., 2014, Petkov et al., 2014). To quantify the differences, the concept of brain network ictogenicity (BNI) was introduced, i.e., a measure of how likely a functional network is of generating seizures *in silico* (Petkov et al., 2014).

For the BNI to be useful for diagnosing people with epilepsy from apparently normal brain activity, it relies on the assumption that the ability of a brain to generate seizures is an enduring feature that should be identifiable during interictal periods. It further assumes that such underlying closeness to seizures is captured by the properties of functional networks. Then, the capacity of a given functional network to generate seizures is assessed by estimating BNI through computer simulations that produce long-term activity from which the volume of epileptiform activity can be evaluated. People with epilepsy are therefore assumed to have resting-state functional networks that are more ictogenic, i.e., that have a higher propensity to generate seizures as estimated by the BNI, compared to healthy people (Schmidt et al., 2016).

Since MEG has the advantage, relative to EEG, of neuromagnetic fields being minimally perturbed by brain tissue, skull and scalp (Supek and Aine, 2016), we expect that MEG-derived functional networks would also be a candidate for the application of BNI. In the current study, we aimed to test whether the BNI concept can be applied to resting-state magnetoencephalography (MEG) data. In particular, we aimed to find whether MEG-derived BNI can differentiate juvenile myoclonic epilepsy (JME) from healthy controls. JME is recognised by the International League Against Epilepsy (ILAE) classification committee as a sub-syndrome of IGE, and makes up by far the largest proportion of IGE in terms of prevalence, alongside Juvenile Absence Epilepsy (JAE), Childhood Absence Epilepsy (CAE) and Epilepsy with Generalised Tonic Clonic Seizures on Awakening (GTCSA) (Engel, 2001, Scheffer et al., 2017). By applying the BNI framework to MEG, positive results would support the generalizability of the BNI across data modalities, over and above the previous EEG applications, and to a specific sub-syndrome of IGE.

## 2 Methods

### 2.1 Participants

We used resting-state MEG data obtained from 26 people with JME and 26 healthy controls. The individuals with epilepsy were recruited from a specialist clinic for epilepsy at University Hospital of Wales in Cardiff, and the healthy individuals were volunteers who had no history of significant neurological or psychiatric disorders. The healthy group was age and gender matched to the epilepsy group. The age range in the epilepsy group was 17 to 47, median 27 years, and in the control group was 18 to 48, median 27 years. There were 7 males in the epilepsy group and 7 males in the control group. Individuals in the epilepsy group had a number of different seizure types and were taking anti-seizure medications (see Krzemiński et al., 2020, Routley et al., 2020 for more details about this dataset). Table 1 summarizes the clinical characteristics of the individuals with epilepsy. This study was approved by the South East Wales NHS ethics committee, Cardiff and Vale Research and Development committees, and Cardiff University School of Psychology Research Ethics Committee. Written informed consent was obtained from all participants.

**Table 1:** Clinical characteristics of the individuals with juvenile myoclonic epilepsy (JME). Age and epilepsy duration are in years, m = male, f = female. Anti-seizure medication (ASM): LEV = levetiracetam, TPM = topiramate, ZNM = zonisamide, LTG = lamotrigine, VPA = valproate, CLB = clobazam, CBZ = carbamazepine. Seizure frequency is in number of seizures per year and is divided in three types of epileptiform activity: MJ = myoclonic jerks, ABS = absence seizures, and GTCS = generalized tonic-clonic seizures. Seizure frequency was based on self-reporting at the time of scan and extrapolated to a number of seizures per year.

ID	Age	Gender	Epilepsy duration	ASM	Seizure frequency		
					MJ	ABS	GTCS
JME1	17.8	f	2.8	LEV	12	365	3
JME2	31.3	f	18.3	TPM, LEV	12	12	1
JME3	27	f	19.0	LEV, ZNM	104	0	1
JME4	20.1	f	3.1	LEV, TPM	0	0	4
JME5	20.7	f	3.7	LTG	4	4	4
JME6	20.4	f	5.4	LTG	12	0	104
JME7	19.2	f	4.2	LEV, ZNM	12	12	12
JME8	20.9	f	12.9	VPA, TPM	104	36	4
JME9	35.3	f	23.3	LTG, CLB	2920	0	1
JME10	30.2	m	16.2	VPA	52	52	4
JME11	23.7	m	8.7	VPA, LEV, CLB	365	2	2
JME12	38.8	f	21.8	VPA, ZNM	365	365	104
JME13	22.2	m	4.2	VPA	104	0	52
JME14	33.1	f	21.1	LEV	12	1	12
JME15	29.7	m	14.7	VPA	12	0	0
JME16	25.7	f	10.7	LEV, ZNM, LTG	6	12	1
JME17	36	f	27.0	LEV, TPM, CLB	0	365	12
JME18	38.6	f	28.6	VPA, LTG	2	0	1
JME19	44.3	m	29.3	VPA, LEV	365	365	2
JME20	47.7	f	40.7	CBZ, LEV, CLB	52	52	1
JME21	26.8	m	8.8	VPA	1	0	0
JME22	22.3	f	10.3	VPA	6	0	1
JME23	38.7	f	24.7	LEV, CLB	0	0	1
JME24	18.9	f	3.9	VPA	0	0	1
JME25	31.1	f	18.1	VPA	1	0	0.2
JME26	22.7	m	10.7	LEV	1	1	0.2

### 2.2 MEG acquisition and pre-processing

MEG data were acquired using a 275-channel CTF radial gradiometer system (CTF System, Canada) at a sampling rate of 600 Hz. We obtained approximately 5 minutes of MEG recordings per individual. The participants were instructed to sit steadily in the MEG chair with their eyes focused on a red dot on a grey background. Each individual also underwent a whole-brain T1-weighted magnetic resonance imaging (MRI) acquired using a General Electric HDx 3 T MRI scanner and an 8-channel receiver head coil (GE Healthcare, Waukesha, WI) with an axial 3D fast spoiled gradient recalled sequence (echo time 3 ms; repetition time 8 ms; inversion time 450 ms; flip angle 20°; acquisition matrix 256 × 192 × 172; voxel size 1 × 1 × 1 mm).

To assess the presence of artefacts and interictal spike wave discharges, the MEG data was divided into 2 s segments and each segment was visually inspected. Artefact-free segments were identified and re-concatenated for each individual. We thus obtained concatenated recordings with a variable length ranging from 204 s to 300 s, and to avoid the potential impact of different recording lengths on our analysis, we only considered the first 200 s of each recording for every individual. The pre-processed data were then filtered in the alpha band (8–13 Hz) and down-sampled to 250 Hz. We focused on the alpha band because it has been shown to be the most informative for differentiating people with epilepsy from healthy controls (Schmidt et al., 2014, Schmidt et al., 2016).

### 2.3 Source mapping from MEG

To infer functional networks from the MEG data, we first mapped the data from the sensor space to the source space. The MEG sensors were co-registered with the structural MRI using the locations of the fiducial coils in the CTF software (MRIViewer and MRIConverter), and we obtained a volume conduction model from the MRI scan using a semi-realistic model (Nolte, 2003). To reconstruct the source signals, we used a linear constrained minimum variance (LCMV) beamformer on a 6-mm template with a local-spheres forward model in Fieldtrip (Oostenveld et al., 2011; http://www.ru.nl/neuroimaging/fieldtrip). We mapped the source signals into the 90 brain regions of the Automated Anatomical Label (AAL) atlas (Hipp et al., 2012). For more details about these methods see our previous studies (Krzemiński et al., 2020, Routley et al., 2020).

### 2.4 Functional networks

We divided the 200-s-long source reconstructed MEG recordings into 10, non-overlapping, 20 s segments. The choice of segment length was motivated by previous studies that aimed to distinguish people with epilepsy from controls using resting-state scalp EEG (Schmidt et al., 2014, Schmidt et al., 2016). For each segment, we computed a functional network using the amplitude envelope correlation (AEC) with orthogonalized signals (Hipp et al., 2012) (see Supplementary Material S1 for more details). We selected this method because it has been shown to be a reliable measure of functional connectivity (Colclough et al., 2016). To remove spurious connections, we generated 99 surrogates from the original MEG signals using the iterative amplitude-adjusted Fourier transform (IAAFT) with 10 iterations (Schreiber and Schmitz, 1996, Schreiber and Schmitz, 2000) (surrogates are randomized time series comparable to the original time series). We excluded connections if their weights did not exceed the 95% significance level compared to the same connection weights as computed from the surrogates (Schmidt et al., 2014, Schmidt et al., 2016, Lopes et al., 2019). Using this method, we obtained 10 functional networks per individual.

### 2.5 Mathematical model

To study the inherent propensity of a MEG functional network to generate seizures, we placed a canonical mathematical model of ictogenicity at each network node, i.e. at each of the 90 brain regions represented in the functional network (Lopes et al., 2017, Lopes et al., 2018, 2019, 2020). The activity of a network node was described by a phase oscillator, which could transit between two states: a ‘resting state’ at which the oscillator fluctuated close to a fixed stable phase and a ‘seizure state’ represented by a rotating phase (see Supplementary Material S2 for more details about the model). This canonical model has been shown to approximate the interaction between neural masses (Lopes et al., 2017).

### 2.6 Brain network ictogenicity

The mathematical model allowed us to generate synthetic brain activity which fluctuated between the resting and the seizure states. To quantify this activity, we used the BNI (Chowdhury et al., 2014, Petkov et al., 2014, Lopes et al., 2017, Lopes et al., 2018, 2019, 2020), which is the average fraction of time that the network spent in the seizure state (see Supplementary Material S3 for more details). We interpret higher values of BNI as representing a higher inherent propensity of the brain to generate seizure activity. Thus, although we used resting-state MEG data to infer the functional networks, we assumed that the underlying brain states may differ in their inherent propensity to generate seizures and this may be captured by our computational framework. We hypothesized that functional networks from JME individuals should be characterized by higher values of BNI than those from healthy individuals.

**Fig. 1:**
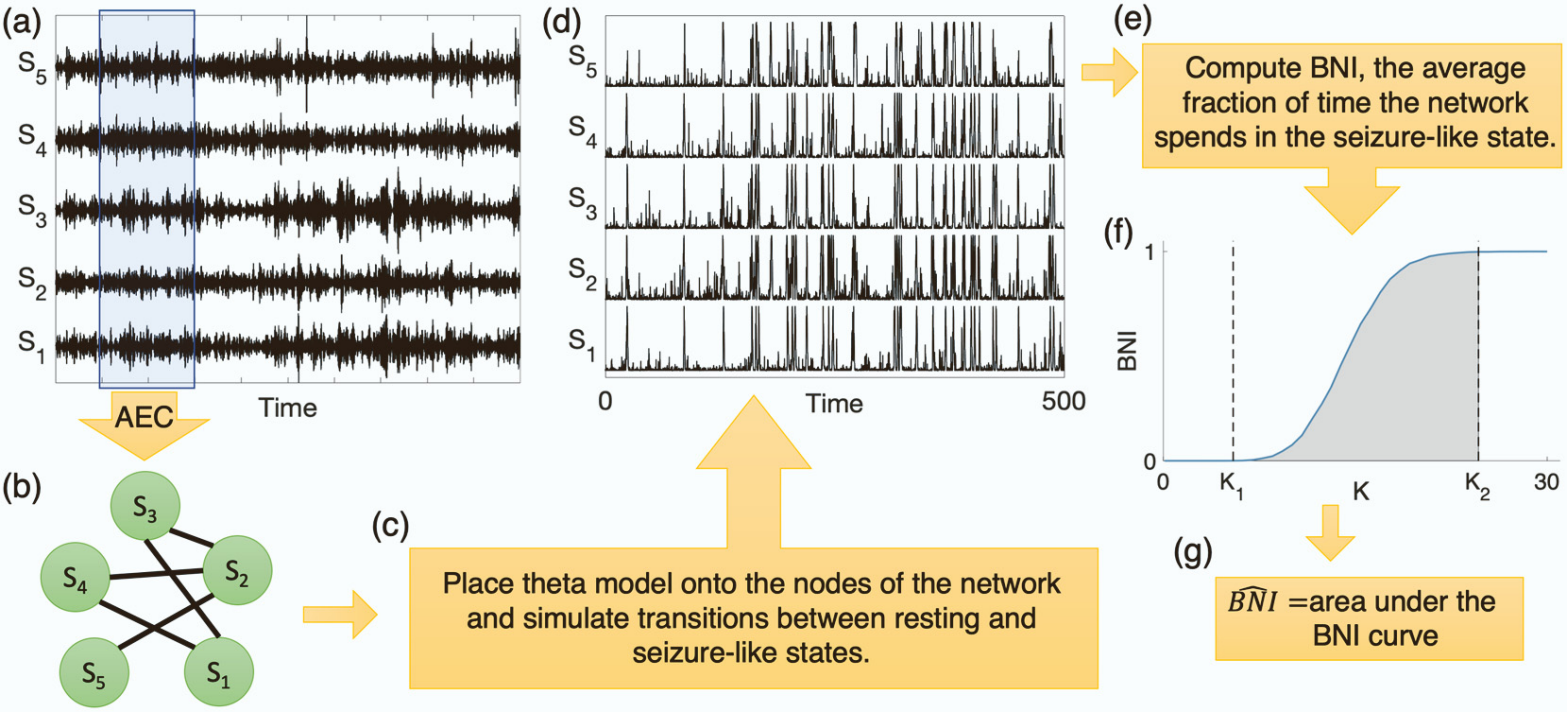
Scheme of the data analysis procedure to compute brain network ictogenicity ($\hat{\mathrm{BNI}}$). (a) We select a magnetoencephalographic (MEG) source reconstructed data segment and by measuring the amplitude envelope correlation (AEC) we obtain (b) a functional network. To assess the propensity of the network to generate seizures, we then use (c) the theta model to simulate (d) synthetic brain activity. We then calculate (e) the brain network ictogenicity (BNI), i.e. the average fraction of time that network nodes spend in seizure-like activity. To avoid an arbitrary choice of K, we compute (f) BNI as a function of K. (g) $\hat{\mathrm{BNI}}$ is then the integral of BNI in the interval $\left[K_{1},K_{2}\right]$, i.e. the area under the BNI curve.

The simulated synthetic activity depends on a model parameter, the global scaling coupling K (see Supplementary Material S2). Higher K values imply stronger neuronal interactions between connected nodes, which in turn leads to higher BNI values. Hence, for a fair comparison of BNI between different functional networks, K must be the same in all simulations. To avoid an arbitrary choice of K, we considered a redefinition of BNI (Lopes et al., 2018). This redefinition consists in computing BNI for a sufficiently large interval of K values in order to capture the full variation of BNI from 0 to 1. Then we calculated $\hat{\mathrm{BNI}}$ as the integral of the BNI in this interval (see Supplementary Material S3). For a meaningful comparison between different functional networks, we used the same interval of K for all simulations. This procedure has been shown to be robust (Lopes et al., 2018). Analogously to the BNI, a higher $\hat{\mathrm{BNI}}$ value corresponds to a higher propensity of a network to generate seizures. Fig. 1 summarizes the key steps of our method.

### 2.7 Statistical methods

We computed 10 functional networks per individual and therefore we obtained 10 $\hat{\mathrm{BNI}}$ values per individual. We then calculated $\langle \hat{\mathrm{BNI}}\rangle$, the average of the 10 $\hat{\mathrm{BNI}}$ values. Finally, we used the Mann-Whitney *U* test to assess whether the median of $\langle \hat{\mathrm{BNI}}\rangle$ was higher in people with epilepsy than in the healthy controls.

## 3 Results

**Fig. 2:**
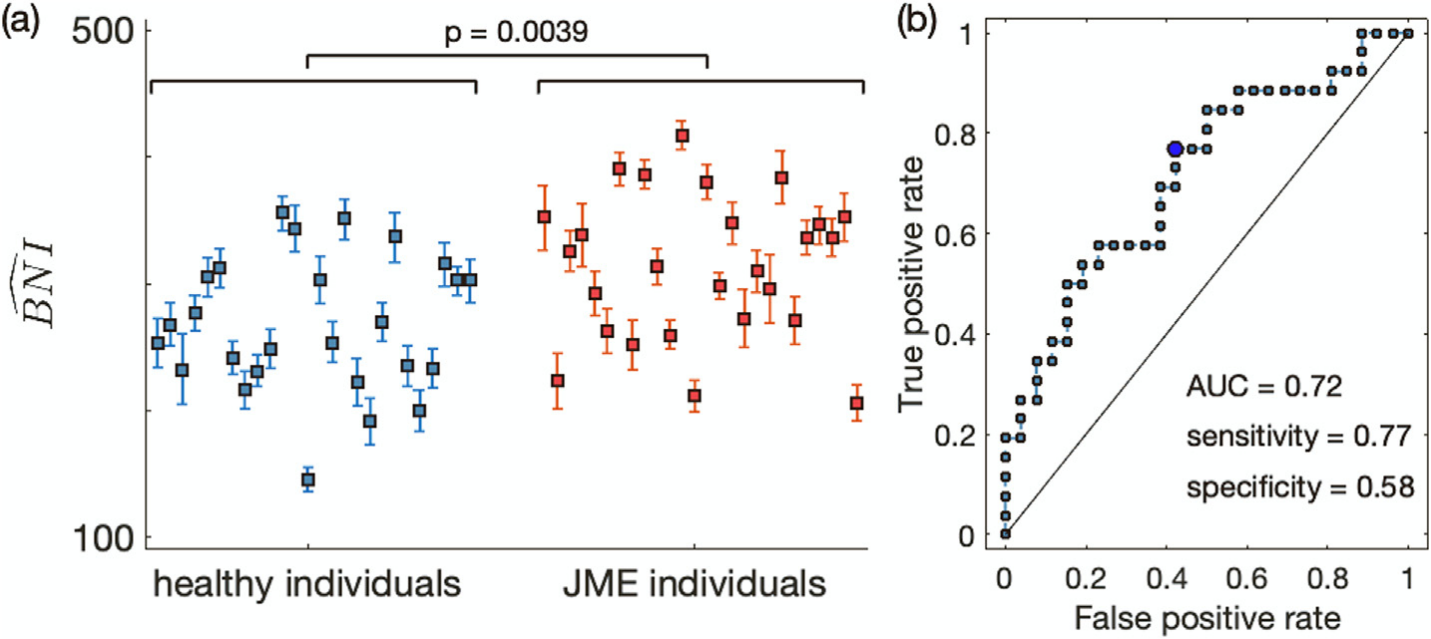
Brain network ictogenicity ($\hat{\mathrm{BNI}}$) in healthy individuals and people with juvenile myoclonic epilepsy (JME). Each marker in panel (a) represents the average $\hat{\mathrm{BNI}}$ (i.e. $\langle \hat{\mathrm{BNI}}\rangle$) of a single individual and the error bars their standard error computed from 10 magnetoencephalographic (MEG) resting-state functional networks. Blue markers correspond to healthy individuals, whereas red markers correspond to individuals with epilepsy. The epilepsy group has a larger $\langle \hat{\mathrm{BNI}}\rangle$ than the healthy group (p=0.0039, Mann-Whitney *U* test). Panel (b) shows the receiver operating characteristic (ROC) curve for one group versus the other using the $\langle \hat{\mathrm{BNI}}\rangle$ as a classifier. The area under the curve (AUC) is 0.72 and the circle identifies the optimal operating point of the ROC curve, for which the sensitivity is 0.77, and the specificity is 0.58.

We considered resting-state MEG recordings from 26 people with JME and 26 healthy controls. To test whether $\hat{\mathrm{BNI}}$ was larger in individuals with JME than in healthy controls, we first built functional networks from MEG source reconstructed data, then we placed a mathematical model of ictogenicity into the network nodes and measured the networks’ propensity to generate seizures *in silico*. Fig. 2(a) shows the $\hat{\mathrm{BNI}}$ for all individuals. Overall, individuals with JME had larger $\hat{\mathrm{BNI}}$ values than healthy controls (p=0.0039, Mann-Whitney *U* test). This finding confirms our hypothesis that resting-state functional networks from people with epilepsy have a higher propensity to generate seizures than those from healthy controls. Note also that for each individual, we observed that $\hat{\mathrm{BNI}}$ had a small variance (i.e. the intraindividual BNI variability is smaller than the interindividual BNI variability), implying that $\hat{\mathrm{BNI}}$ was consistent across the 10 different MEG resting-state functional networks of each individual. We then tested whether $\hat{\mathrm{BNI}}$ could be used for individual classification as to whether individuals had epilepsy. Fig. 2(b) shows the receiver operating characteristic (ROC) curve. The area under the curve (AUC) was 0.72, the sensitivity was 0.77, and the specificity was 0.58. The $\hat{\mathrm{BNI}}$’s classification accuracy was 73%.

The results in Fig. 2 may be confounded by a number of factors. Namely, epilepsy duration and seizure frequency may have an impact on the $\hat{\mathrm{BNI}}$ values. Figure S1 shows the $\hat{\mathrm{BNI}}$ versus these clinical characteristics in the JME group. From visual inspection, the figure suggests that while individuals with short epilepsy duration or low seizure frequency may exhibit both low and high $\hat{\mathrm{BNI}}$ values, individuals with relatively longer epilepsy duration (larger than 20 years) and higher seizure frequency (higher than 200 seizures per year) present high $\hat{\mathrm{BNI}}$ values. We also computed the Pearson’s correlation between the $\hat{\mathrm{BNI}}$ values and the seizure frequency of each seizure type (myoclonic jerks, absence seizures, and generalized tonic-clonic seizures), as well as the total seizure frequency across the three types. The correlations were not significant (r<0.29 and p>0.15 in all tests).

## 4 Discussion

To date, the BNI framework has proved to be valuable for epilepsy diagnosis using scalp EEG in IGE (Schmidt et al., 2014, Schmidt et al., 2016, Petkov et al., 2014), assessment of epilepsy surgery using intracranial EEG in focal epilepsy (Goodfellow et al., 2016, Laiou et al., 2019, Lopes et al., 2020, Lopes et al., 2018, Lopes et al., 2017), and epilepsy classification using scalp EEG (Lopes et al., 2019). Here we extended previous results, testing whether the concept of BNI could differentiate people with JME from age and gender matched healthy controls using resting-state MEG data. We found that the BNI is on average higher in the JME group than in the control group. We further found that, as a classifier, the BNI yields a sensitivity of 0.77, a specificity of 0.58, and an AUC of 0.72. This classification performance is similar to previous results in classifying people with IGE using scalp EEG (Schmidt et al., 2016). However, it is worth noting that our results from people with JME may not be directly comparable to those in the study by Schmidt et al. that were based on an IGE cohort that would have included other sub-syndromes, JAE, CAE and GTCSA, in addition to JME. Nevertheless, if we assume the existence of common traits across the IGE spectrum (including JME), our findings may suggest the hypothesis that MEG and scalp EEG may yield similar diagnostic power through the BNI framework, despite MEG often being considered superior to EEG in recording reliable brain signals (Supek and Aine, 2016). More generally, our results further consolidate the validity and usefulness of quantifying resting-state functional networks using a mathematical model of seizure transitions to assess the propensity of the brain to generate seizures.

Resting-state MEG functional networks have been previously shown to be altered in people with epilepsy relative to healthy controls (van Dellen et al., 2012, Niso et al., 2015, Hsiao et al., 2015, Wu et al., 2017, Routley et al., 2020). For example, Niso et al. (2015) used 15 graph-theoretic measures to quantify resting-state MEG functional networks from people with frontal focal epilepsy, generalized epilepsy and healthy individuals. They found that functional networks from generalized epilepsy had greater efficiency and lower eccentricity than those from controls, whereas functional networks from frontal focal epilepsy exhibited only reduced eccentricity over fronto-temporal and central sensors relative to networks from controls. Furthermore, machine learning has been used to also differentiate people with epilepsy from controls (Soriano et al., 2017). Our study distinguishes from these studies by not only searching for differences between functional networks in health and disease, but instead test a specific mechanistic hypothesis that justifies the difference. Thus, our approach is more readily interpretable and may offer insight into why altered functional networks underlie epilepsy.

We acknowledge that our study has some limitations. First, in order to truly test how MEG-based predictions compare to scalp EEG-based predictions, we would need both MEG and EEG data collected from the same participants. Future work should assess whether predictions based on both data modalities would deliver equivalent individual classification. Second, people with JME were taking anti-seizure medication, which may have potentially reduced the BNI in some JME individuals, making them indistinguishable from healthy individuals. Future studies should consider newly diagnosed drug-naïve individuals. This may be particularly important to also control for the effect of epilepsy duration and seizure frequency on BNI. Our results suggest that individuals with longer epilepsy duration and higher seizure frequency were more likely to be characterized by high BNI. On one hand this is an expected observation, i.e. BNI should be higher for individuals more prone to seizures and also those for which a longer disease may have had an impact on resting-state functional connectivity. On the other hand, these were individuals for which diagnosis could be less challenging. Third, we focused our analysis on differentiation of people with JME from healthy controls. We therefore cannot exclude the possibility that our findings are specific to JME. More comprehensive datasets will be needed to explore whether our findings generalize to other types of epilepsy.

## 5 Conclusions

By extending the application of the BNI framework to MEG, our results demonstrate that the BNI can be useful to interrogate different data modalities beyond EEG. We showed that resting-state MEG from people with JME is characterized by higher BNI than that from healthy controls. Our results suggest that BNI applied to resting-state MEG may aid in the diagnosis of JME.

## Declaration of Competing Interest

JT is co-founder and Director of Neuronostics.
