# Supplementary material for: A computational biomarker of juvenile myoclonic epilepsy from resting-state MEG
Source: Clin Neurophysiol. 2021 Apr;132(4):922–7. doi: 10.1016/j.clinph.2020.12.021 (PMC7992031; doi:10.1016/j.clinph.2020.12.021)
Supplement: Supplementary data 1 [file mmc1.docx]

**Supplementary Material**

**Text S1: Amplitude envelope correlation**

To construct functional networks from the MEG source reconstructed data, we used the amplitude envelope correlation (AEC) with orthogonalized signals (Hipp et al., 2012).

To compute the AEC between pairs of source signals, we first orthogonalized the source signals to avoid spurious correlations due to source leakage (Hipp et al., 2012). The orthogonalization is given by

$$\begin{aligned} Y_{\perp X}\left( t,f \right)=\mathrm{imag}\left( Y\left( t,f \right)\frac{X^{*}\left( t,f \right)}{\left| X\left( t,f \right) \right|} \right),\#\left( S1 \right) \end{aligned}$$

where $X\left( t,f \right)$ and $Y(t,f)$ are two source signals in a frequency band $f$, $X^{*}\left( t,f \right)$ is the complex conjugate of $X\left( t,f \right)$, $Y_{\perp X}\left( t,f \right)$ is the orthogonalized $Y(t,f)$ with respect to $X(t,f)$, and $\mathrm{imag}(x)$ stands for the imaginary part of $x$. The orthogonalization is also done in the opposite direction, from $Y(t,f)$ to $X(t,f)$, yielding $X_{\perp Y}\left( t,f \right)$. We then calculated the Pearson’s correlation between $Y_{\perp X}\left( t,f \right)$ and $X(t,f)$, and between $Y(t,f)$ and $X_{\perp Y}\left( t,f \right),$ and took the average of the two values to make the AEC symmetric.

**Text S2: Theta model**

The dynamics of the network oscillators $\theta_{i}$ was described by the theta model (Lopes et al., 2017, 2018, 2019, 2020):

$$\begin{aligned} \dot{\theta_{i}}=\left( 1-\cos\theta_{i} \right)+\left( 1+\cos\theta_{i} \right)I_{i}\left( t \right),\#\left( S2 \right) \end{aligned}$$

where $I_{i}\left( t \right)$ is the input current received by node $i$ at time $t$, accounting for noise and the interaction with other oscillators in the network:

$$\begin{aligned} I_{i}\left( t \right)=I_{0}+\xi^{\left( i \right)}\left( t \right)+\frac{K}{N}\sum_{i\neq j} a_{ji}\left[ 1-\cos\left( \theta_{j}-\theta^{\left( s \right)} \right) \right],\#\left( S3 \right) \end{aligned}$$

where $I_{0}+\xi^{\left( i \right)}\left( t \right)$ is Gaussian noise, $K$ is a global scaling factor of the network’s interaction, $N$ is the number of nodes ($N=90$), $a_{ji}$ is the $j,i$^th^ entry of the weighted adjacency matrix representing the functional network, and $\theta^{\left( s \right)}$ is the fixed stable phase for an isolated oscillator (Lopes et al., 2017). The noise represents signals coming from remote brain regions outside of the functional network under consideration. The input current defines whether a node is at the resting state ($I_{i}<0$) or at the seizure state ($I_{i}>0$). A saddle-node on invariant circle (SNIC) bifurcation at $I_{i}=0$separates the two states. Parameters were chosen according to previous studies (Lopes et al. 2017, 2018, 2019, 2020): $I_{0}=-1.2$ and noise standard deviation $\sigma=0.6$. The global scaling factor $K$ was the only free parameter.

**Text S3: Brain network ictogenicity (BNI)**

The BNI is the average fraction of time that nodes spend in the seizure state (Chowdhury et al., 2014; Petkov et al., 2014; Lopes et al., 2017, 2018, 2019, 2020), i.e.

$$\begin{aligned} \mathrm{BNI}(K)=\frac{1}{N}\sum_{i=1}^{N} \frac{t_{sz}^{\left( i \right)}\left( K \right)}{T}, \#\left( S4 \right) \end{aligned}$$

where $t_{sz}^{\left( i \right)}(K)$ is the time that node $i$ spent in the oscillatory state for a given choice of $K$, during the simulation time $T$. We used $T=4\times{10}^{6}$ time steps, and the seizure state was defined as any activity larger than a threshold as described in (Lopes et al., 2017).

To avoid an arbitrary choice of $K$, we use a redefined $\mathrm{BNI}$ (Lopes et al., 2018) given by

$$\begin{aligned} \hat{BNI}=\int_{K_{1}}^{K_{2}} \mathrm{BNI}\left( K \right)dK, \#\left( S5 \right) \end{aligned}$$

where $K_{1}$ and $K_{2}$ are chosen such that one can capture the full variation of $BNI$ from 0 to 1. In order to assess the $\hat{BNI}$ from all functional networks, we first plotted all $BNI(K)$ curves together and chose a sufficiently large interval $[K_{1},K_{2}]$ that comprised the variation from 0 to 1 for all curves. We used the same interval $\left[ K_{1},K_{2} \right]=[5,500]$ for all networks and individuals.

**Supplementary Figure**

**
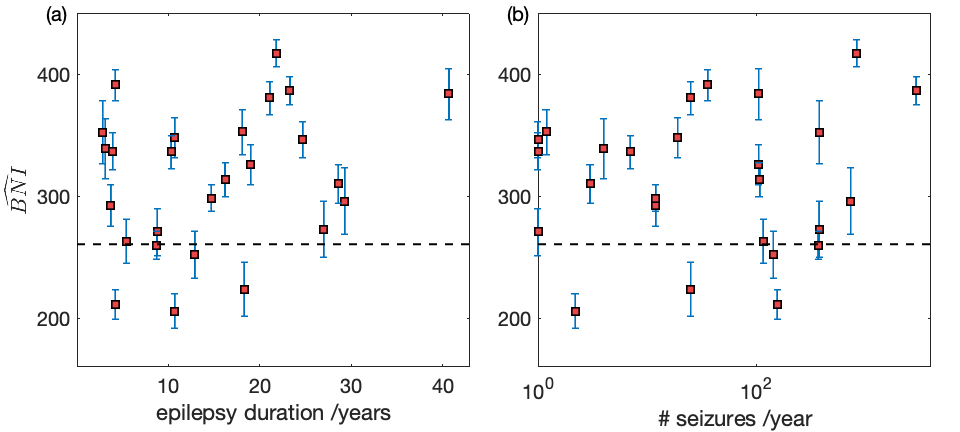
**

**Figure S1.** Brain network ictogenicity ($\hat{BNI}$) in people with juvenile myoclonic epilepsy (JME) as a function of (a) epilepsy duration and (b) seizure frequency. Each marker represents the average $\hat{BNI}$ (i.e. $\left\langle\hat{BNI} \right\rangle$) of a single individual and the error bars their standard error computed from 10 magnetoencephalographic (MEG) resting-state functional networks. Seizure frequency is defined as the number of seizures per year self-reported by each individual (the number of seizures includes absence seizures, generalized tonic-clonic seizures and also myoclonic jerks, see Table 1). Note that the seizure frequency is presented in a logarithmic scale. The horizontal dashed line is the median $\left\langle\hat{BNI} \right\rangle$ of the healthy group, which is represented here to clarify which markers correspond to $\left\langle\hat{BNI} \right\rangle$s higher than those in the healthy group.
